# Building a Population Representative Pediatric Biobank: Lessons Learned From the Greater Cincinnati Childhood Cohort

**DOI:** 10.3389/fpubh.2020.535116

**Published:** 2021-01-14

**Authors:** Lisa J. Martin, Liza Bronner Murrison, Melinda Butsch Kovacic

**Affiliations:** ^1^Division of Human Genetics, Department of Pediatrics, Cincinnati Children's Hospital Medical Center, University of Cincinnati School of Medicine, Cincinnati, OH, United States; ^2^Division of Asthma Research, Department of Pediatrics, Cincinnati Children's Hospital Medical Center, University of Cincinnati School of Medicine, Cincinnati, OH, United States; ^3^Department of Rehabilitation, Exercise and Nutrition, Sciences, College of Allied Health Sciences, University of Cincinnati, Cincinnati, OH, United States

**Keywords:** children, research, epidemiology, community, genetics

## Abstract

**Background:** Biobanks can accelerate research by providing researchers with samples and data. However, hospital-based recruitment as a source for controls may create bias as who comes to the hospital may be different from the broader population.

**Methods:** In an effort to broadly improve the quality of research studies and reduce costs and challenges associated with recruitment and sample collection, a group of diverse researchers at Cincinnati Children's Hospital Medical Center led an institution-supported initiative to create a population representative pediatric “Greater Cincinnati Childhood Cohort (GCC).” Participants completed a detailed survey, underwent a brief physician-led physical exam, and provided blood, urine, and hair samples. DNA underwent high-throughput genotyping.

**Results:** In total, 1,020 children ages 3–18 years living in the 7 county Greater Cincinnati Metropolitan region were recruited. Racial composition of the cohort was 84% non-Hispanic white, 15% non-Hispanic black, and 2% other race or Hispanic. Participants exhibited marked demographic and disease burden differences by race. Overall, the cohort was broadly used resulting in publications, grants and patents; yet, it did not meet the needs of all potential researchers.

**Conclusions:** Learning from both the strengths and weaknesses, we propose leveraging a community-based participatory research framework for future broad use biobanking efforts.

## Introduction

Biobanks allow storage of biological specimens and corresponding data for biomedical research, particularly omics studies (1). While providing convenient access to human samples, sample utility can be variable. Biobanks relying on residual sample may have limited sample types and accompanying data. Further, participants in hospital-based biobanks may not be population representative. These differences introduce possible confounding (2, 3).

Population-based biobanks offer an alternative. Population sampling ensures subjects are recruited from the same source as cases (4). A population-based approach can capture specific racial/ethnic populations that may be underrepresented (5). As racial/ethnic minorities make up nearly 40% of the US population (6), minority inclusion is critical for clinical research generalizability (5, 7), healthcare equity (8, 9), and ancestry/ethnicity-specific analyses (10). Unfortunately, costs and poor participation often precludes such sampling for independent researchers (4).

To address these issues, a group of researchers at Cincinnati Children's Hospital Medical Center (CCHMC) led an initiative supported by institutional funds to create a pediatric population-based representative biobank entitled the “Greater Cincinnati Childhood Cohort (GCC).” The goal of the study was to collect a representative sample of the Greater Cincinnati Metropolitan children for use in clinical and translational studies.

## Methods

### Development of Institutional Initiative

In 2006, a meeting was held to discuss research projects that might benefit from an institutional cohort gauging interest from faculty involved with numerous rare and common conditions. Based on the broad interest, Cincinnati Children's Research Foundation decided to fund a pediatric biobank for the greater good of its research endeavors.

### Sampling Design

The goal was to obtain a population representative sample which would benefit research endeavors. While many sampling designs were considered, recruitment based on race, sex, age, and income strata representative of the Greater Cincinnati area according to the 2000 and 2005 United States (US) Census population estimates was chosen.

Inclusion criteria were as follows: participants were between the ages of 3 and 18 years (prior to 18th birthday) at the time of enrollment living in Greater Cincinnati [Hamilton (OH), Clermont (OH), Butler (OH), Warren (OH), Boone (KY), Kenton (KY), Campbell (KY)]. Parents/guardians provided permission through written informed consent. This consent requested permission for use of the survey and exam data and the samples (including DNA) for ongoing and future studies related to the health and well-being of children. Children aged 11 years or greater provided written assent. Once a child turned 18 years of age, they were sent a letter which would allow them to cease participation. Exclusion criteria included participating in an investigational study in the past thirty days and having a parent reported diagnosis of a genetic syndrome. One participant per family was allowed to participate. Prior to study initiation, the study was presented to and received feedback from our Patient Advisory Council. This cohort was approved by Cincinnati Children's Hospital Medical Center's Institutional Review Board.

### Recruitment

Recruitment for the GCC was multi-faceted. First, participants of a previously established early childhood study were invited. Second, flyers were placed on research study notification boards at CCHMC as well as shared with ~20 community organizations including elementary schools (flyers sent home with children), social service agencies, day cares, summer camps, at a church and at health fairs. Our data suggested that the most participants heard about the study from friends of CCHMC patients.

Potential subjects were screened by telephone to ensure eligibility. US Census tract monitoring ensured cohort diversity and representativeness (an equal number of males and females, and ~85% white non-Hispanic, 12% African-American, and 3% Asian, Hispanic and other minorities, which represented the population distribution of the Greater Cincinnati Metropolitan area). Within these racial categories, an equal number of males and females in each of 5 age groupings (age under 6, ages 6 to <9, ages 9 to <12, ages 12 to <15, ages 15 to <18 years) was considered the target enrollment. Parental-reported household income of participants was also tracked to ensure that the biobank reflected the metropolitan area (20% of the participants with household income < $20,000, 25% of the participants with incomes between $20,000 and $39,999, 31% of participants with incomes between $40,000 and $74,999, and 24% of participants with incomes of at least $75,000). Review of population representativeness was carried out bimonthly and marketing was modified to target specific strata.

### Study Procedures

Participants were mailed the informed consent document after phone screening. At the study visit, study staff reviewed the informed consent document with the participant and parent/legal guardian. A questionnaire administered by a clinical research coordinator captured information on demographics, family/home environment, medical history, and medication use. A licensed physician performed a brief physical exam.

Height, weight, waist and hip circumferences, blood pressure, and heart rate were collected in duplicate or triplicate if notable differences were observed. Anthropometrics were collected following a protocol used by CCHMC investigators (11). To ensure consistency between repeated measures, the coefficient of variation between the measures for each subject was calculated; most exhibited strong consistency (CV <5%). When CV > 5% among triplicate measures, the two values which yielded the CV <5% were averaged, otherwise the observation was set to missing. Averages were used in the final analyses. Body mass index (BMI) was calculated as weight(kg)/height(m)^2^; age and sex adjusted percentile and z-scores were determined based on CDC 2000 growth curves (12).

Serum, plasma and whole blood were collected to allow for DNA isolation, RNA isolation, clinical diagnostic and biomarker analysis. Urine and hair were collected and stored in sterile non-contaminating receptacles.

### Data Management and Access

Phenotypic data was housed in SQL. Projects which use only de-identified data are reviewed at the Director/co-Director level for scientific merit. Projects which use personal health information or samples are reviewed by a Scientific Advisory Committee, made up of 12 faculty researchers spread across multiple divisions within the Department of Pediatrics. Investigators using the GCC data were expected to consult with the IRB with respect to their specific projects prior to obtaining data.

### Data Analysis

The cohort was described using mean ± standard deviation or frequencies. To test whether there were differences in participant characteristics by race, whites and black participants were compared using chi-square goodness of fit tests or *t*-tests. To determine if the population was representative of the general catchment area, 2010–2012 3 year estimates for the Greater Cincinnati Metropolitan area from the US Census American Communities Survey and national estimates were used (13). GCC publications were entered into SCOPUS to determine article metrics.

## Results

### Characteristics of the Cohort

**Figure 1:**
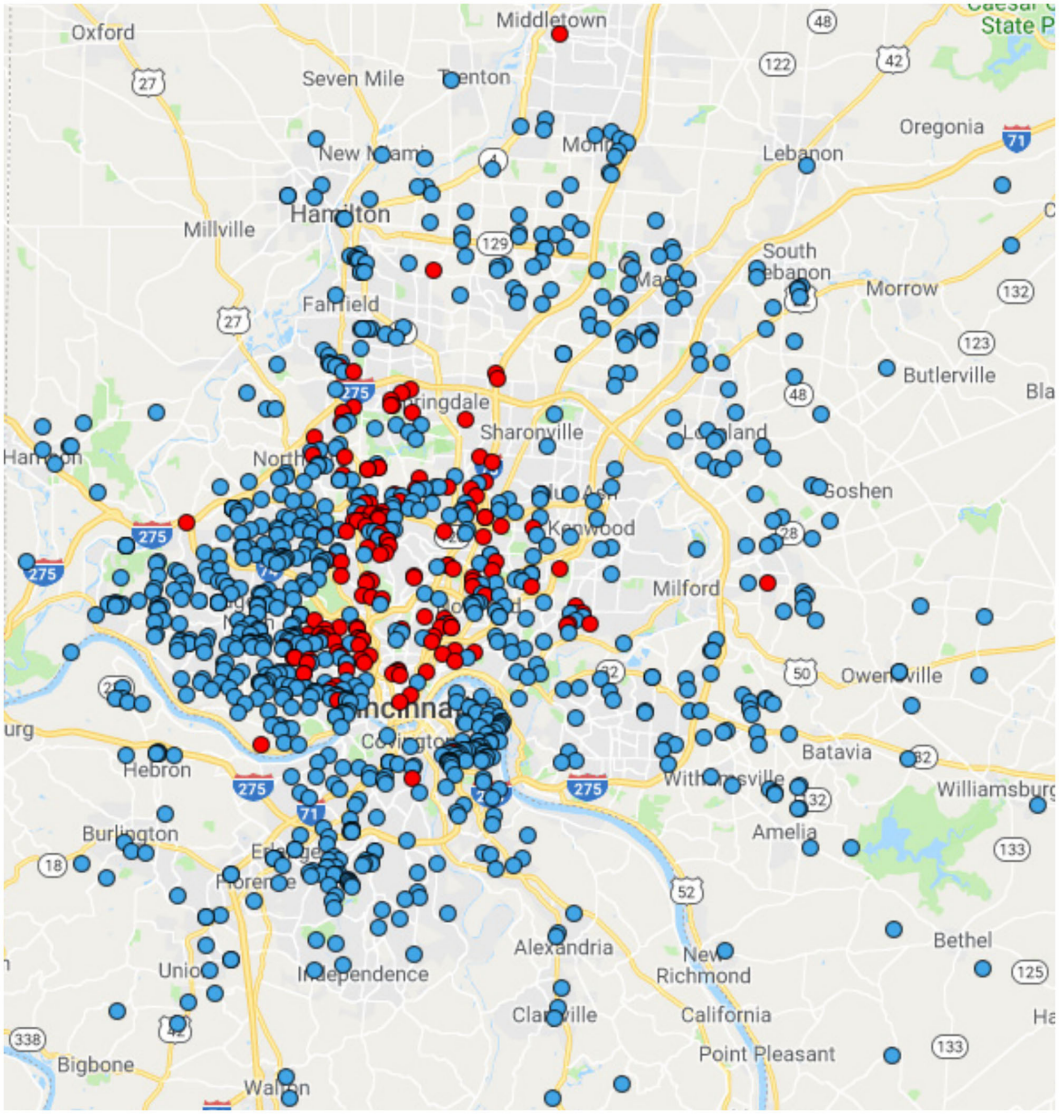
Geographic distribution of the GCC Participants. Blue dots represent individuals who self-report as white. Red dots represent individuals who self-report as black.

Between 2007 and 2011, 1,020 children were enrolled (Figure 1). The cohort was 84% non-Hispanic white, 15% non-Hispanic black, and 2% other race or Hispanic (Table 1). Age and sex were distributed similarly between whites and blacks (*p* > 0.05). Blacks had lower parental education (*p* < 0.0001), lower income (*p* < 0.0001), and lower rates of private insurance (*p* < 0.0001). Racial distribution of the GCC were similar to Greater Cincinnati Metropolitan area and the United States (Supplementary Figure 1).

**Table 1:** Description of the demographic characteristics of the participants.

	**White**	**Black**	**Other^**a**^**	***P*-value (White vs. Black)**
Frequency (*N*)	852	150	18	–
Sex (% M)	49.1	50.0	33.3	0.83
Age (years ± SD)	11.0 ± 4.2	10.6 ± 4.2	12.4 ± 4.1	0.27
Education (Parents)				<0.0001^*^
No high school diploma (%)	1.7	8.8	0	
High school diploma (%)	8.3	16.3	11.1	
Some college (%)	14.9	38.8	27.8	
2 year degree/technical (%)	16.3	21.8	16.7	
Bachelor's degree or more (%)	58.8	14.3	44.4	
Annual family income				<0.0001^*^
Under $10K (%)	3.9	24.0	16.7	
$10K – 19,999 (%)	3.2	20.0	5.6	
$20K – 29,999 (%)	4.2	17.7	5.6	
$30K – 39,999 (%)	6.7	14.0	5.6	
$40K – 49,999 (%)	6.5	4.0	11.1	
$50K – 74,999 (%)	26.4	12.7	27.8	
$75K – 99,999 (%)	22.7	6.0	5.6	
$100K or more (%)	26.4	2.7	22.2	
Insurance				<0.0001^**^
Private (%)	85.2	36.0	66.7	
Public (%)	12.1	62.0	27.8	
No insurance (%)	1.9	2.0	5.6	
Both Public and Private (%)	0.7	0	0	

a Others include Hispanic and multi-racial study participants.

* Comparison of ordinal ranking using Wilcoxon rank sum.

** *Comparison of private only vs. other*.

Overall, 659 (64.6%) participants reported a medical condition, with the most common conditions being injuries (26.2%), tonsillectomy and adenoidectomy (12.7%), and ear tubes (9.5%). Health status with respect to common conditions differed by race, with blacks having higher rates of obesity, asthma, and very low birth weight (under 2500 g) than whites (Table 2).

**Table 2:** Description of the health status of participants.

	**White (*n* = 852)**	**Black (*n* = 150)**	**Other^**a**^ (*n* = 18)**	***P*-value (White vs. Black)**
**Obesity**				
BMI z -scores(STD)	0.40 ± 0.96	0.69 ± 1.04	1.2 ± 0.94	0.0017
Overweight %	14.8	15.5	22.2	0.015
Obese %	10.4	18.9	38.9	
**Allergic conditions**				
Asthma %	12.7	24.0	33.3	0.0006
Eczema %	17.5	40.7	38.9	<0.0001
Environmental allergies %	41.5	37.3	38.9	0.33
**Birth conditions**				
Prematurity %	10.8	12.0	5.6	0.67
Birth weight under 2500g %	5.7	12.8	0	0.0034

a Others include Hispanic and multi-racial study subjects.

BMI z scores and percentiles were calculated using CDC growth charts; people with BMI percentile ≥85% are considered overweight.

*STD, standard deviation*.

### Cohort Utilization

**Figure 2:**
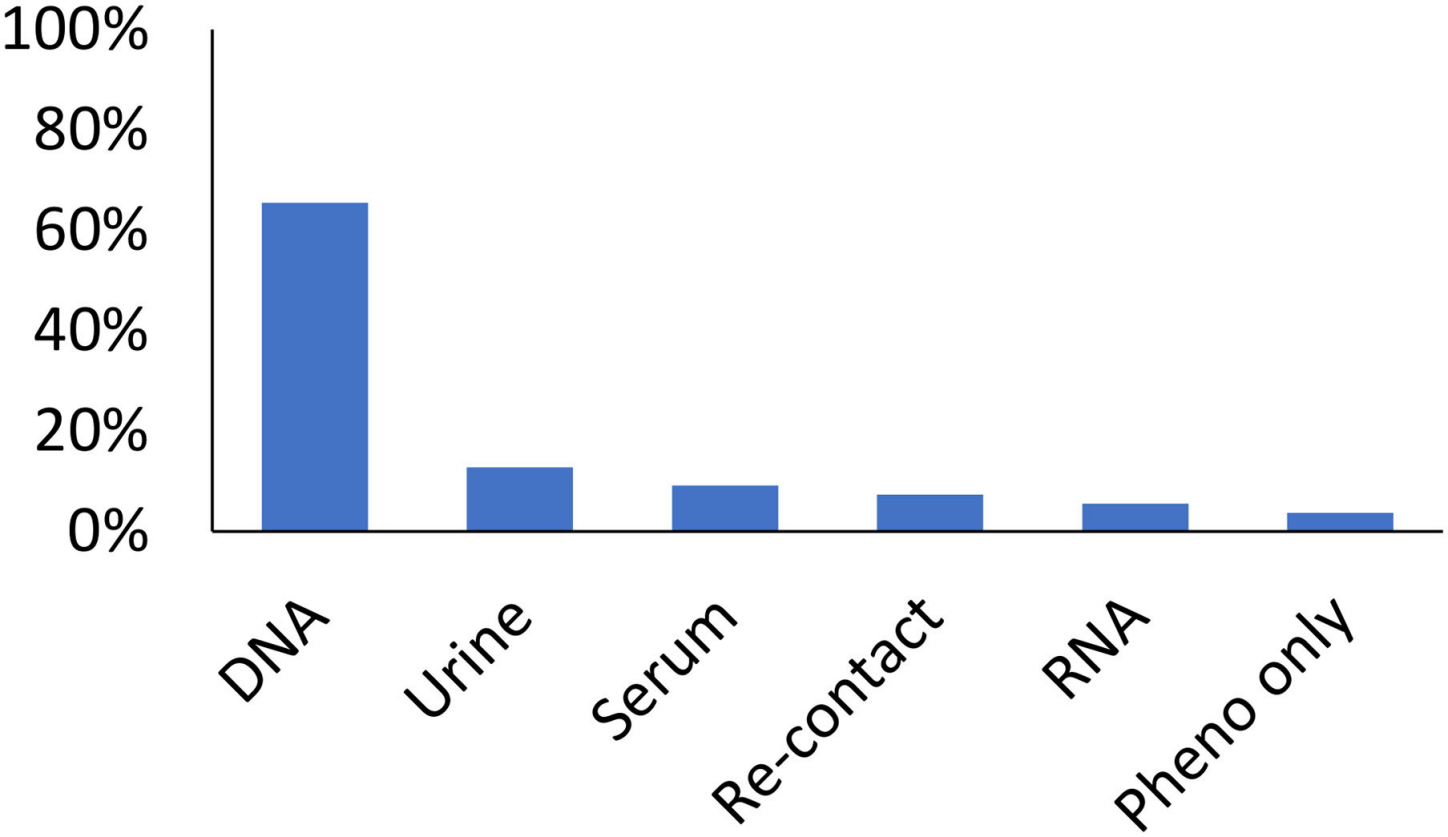
Utilization of the GCC by type of sample by the 65 studies which requested data. Bars represent the proportion of studies utilizing a sample/data. Studies may have utilized multiple types of data/sample.

From 2007 to 2018, there were 65 projects which requested data and/or samples (Figure 2) resulting in 60 publications (14–73), a patent application, and 9 externally funded grants. These projects focused on a diverse set of phenotypes including arthritis, asthma, epilepsy, obesity, and lupus. Most projects (59.6%) had a genetic component. Slightly over one third of projects used bio-specimens (serum, PBMC, or urine). Just under 5% of the studies only used phenotype data. The lead investigators for the projects included 39 individuals from 17 divisions across CHMC. For the publications, 161 authors were listed, with the median number of publications being 2 per author (interquartile range 2–3; maximum 20). Among these publications, the h-index was 31 with 2,596 citations. Publication major research areas included medicine (77%), immunology and microbiology (41%), and biochemistry, genetics, and molecular biology (36%). With respect to grant funding (from 6 PIs in 5 divisions), 6 of the grants were program project or U-series grants demonstrating the wide impact of the GCC.

### Benefits of the Cohort

There were two common reasons researchers reported using the GCC. First, researchers noted need for a highly specific group of controls or samples collected in a specific manner. Many required samples from children free from specific a disease or co-morbid condition. Several researchers noted challenges in using publicly available data which did not have required information or had substantially different population distributions compared to their cases. Moreover, researchers noted that residual diagnostic samples were often not collected in a sufficient manner or did not have the necessary accompanying data. Students and trainees noted funding and time limitations for de novo collection.

### Limitations of the Cohort

In managing the cohort, several limitations were noted. First, the sample size for non-white participants was insufficient for genetic studies. Second, the cross-sectional design is a missed opportunity as a child's health status is dynamic. Third, some investigators were unable to use the resource because of sample requirements or data availability. Fourth, some data and samples (hair) were never used, wasting resources. Fifth, while there was a diverse group of investigators who used the data, this user group represented a small fraction of potential users. Lastly, participant communication was minimal, limited to annual birthday cards and holiday cards unless re-contact was medically indicated. In later years, a newsletter was sent to highlight the cohort's successes and thank subjects for participation.

## Discussion

Meeting the needs of a diverse set of investigators is a central premise for biobanks. When planning the Greater Cincinnati Childhood Cohort (GCC), an institutionally-supported biobank, investigators sought to enable broad use across a wide variety of studies. Recognizing the bias in recruiting only patients seeking care within the hospital, investigators employed a population-based design. Our experiences with the GCC after 10 years have allowed us to consider the benefits and challenges. The wide-ranging cohort utilization and return on investment is evidence of success. However, limitations included minimal racial diversity and static design. Seeking to maximize benefits and minimize challenges for future endeavors, we consider leveraging community-based participatory research approaches.

### Strengths of the GCC and Population Representative Biobanks

Over its lifespan, the GCC has advanced a diverse set of research projects as evidence through publications and grants. GCC's success can be attributed to three factors. First, while not as large as many biobanks, a distinct advantage was consistent phenotyping and non-biased recruitment. Having complementary phenotypic data and biological samples is beneficial (74–76). GCC participants were not necessarily free of disease, as to be expected with population recruitment. GCC participants had similar rates of prematurity, but higher rates of asthma and obesity compared to US estimates (77–80). The presence of disease in population-based controls highlights the need to systematically capture phenotypes, especially for common diseases.

Second, there were a diverse set of samples. While a major motivation for GCC's initiation was to serve as a resource for genetic studies, many projects were not genetics focused. Our collection of samples beyond blood, is not typical for most biobanks where the majority of have only collected serum or plasma (77%), few have collected urine (30%) or hair (3%) (81). Paired samples further expand utility.

Third, the GCC cohort was designed to benefit a broad array of research endeavors. While many researchers required a subset of the data, recruitment of participants who could be used in multiple studies was cost effective. Currently, the approach to develop a general institutional research resource is less common than disease focused approaches. Indeed, only 29% of biobanks are developed to facilitate a broad spectrum of research (81).

### Limitations of the GCC

While the GCC exhibited many benefits, there were also limitations. First, as the GCC was designed to be population-based in a region that is predominantly white, it had limited numbers of minority participants. This is a problem because blacks are more likely to reside in under-resourced neighborhoods and to experience health disparities (82–87). As inclusion of minority subjects and mitigating health disparities is increasingly a priority of researchers, biomedical research studies, therefore, must ensure that racial diversity is captured (88). Since there are many barriers to participation by racial/ethnic minorities (89), over sampling of minority participants may be needed. Indeed, “All of Us,” NIH's precision medicine initiative, and NHANES oversample minorities to ensure sufficient representation (90).

Although the protocol allowed recall and follow-up of cohort participants, the lack of resources supporting the cohort's follow-up resulted in having a cross-sectional design. While detailed phenotypic information was collected, health status is dynamic with some conditions not diagnosed until later ages. For example, tests for asthma diagnosis are difficult to perform in children under six (91). Likewise, obesity status may change through childhood (92). The cross-sectional nature of the cohort means that the only samples/data available are the ones collected from a single point in time. Continuously evolving technological advances such as genome editing (93) and metabolomics (94) cannot be applied to GCC samples. Further, some data and samples have yet to be used, reducing return on investment.

Lastly, participant outreach was minimal. Specifically, no research findings were shared. This is a problem as one of the seven ethical principles of research is respect for subjects which includes having a mechanism to inform participants of the impact of their participation (95). Unfortunately, dissemination of aggregate research results occurs infrequently (96), even though research participants state that they want such results (97, 98). Further, in addition to aggregate research results, providing education, and sharing clinically relevant individual results to study participants would encourage continued participation. Additionally, when using data/samples, there was no mechanism to consider the participants point of view. While research participant advisory groups often are used when designing studies, inclusion of participant advocates on the advisory committee could provide additional participant protections.

### Starting Over: Design a Generalizable Resource for Biomedical Research

Given the benefits and limitations, biobanks that capture generalizable information are essential. Prior work has demonstrated challenges in capturing community diversity using hospital based recruitment (99, 100). However, population representative sampling may lead to insufficient minority numbers. To overcome these issues, we propose to work with community partners to establish a research registry serving as a resource for researchers looking for data and samples for studies, sharing relevant clinical information between healthcare information exchanges or directly with participants, sharing aggregate data with both participants and community partners (e.g., via tailored reports, websites, and newsletters), and spawning community-based participatory research (CBPR) efforts (101). CBPR benefits include increased trust, improved data quality and validity due to participant input, and enhanced data relevance given the input from diverse vested individuals (102). Notably, CBPR consistently increases research participation in under-resourced populations (103–105).

CPBR designs benefit both the community and researchers. Community members gain empowerment and the opportunity to gain understanding on matters important to them. Researchers gain access to a population open to research, an infrastructure for sample and data collection, as well as feedback on the relevance of questions. To ensure a mutually beneficial relationship, researchers would be expected to share results with the community and individual participants as appropriate, providing value for the community. To maximize the utility of the resource, researchers would also be expected to share the data with the biobank, facilitating capacity building. Including a researcher-led component yields dynamic data and sample collection, driven by research questions to ensure maximal utility. Further, following pediatric biobank participants into adulthood should be considered.

While CBPR is an attractive approach, substantial upfront investment is required. Forging partnerships can be challenging (102). Academic partners must create an environment where community members feel comfortable raising issues, sharing opinions, and asking questions. Another challenge is communication. Many under-resourced populations have lower health literacy levels (106–109). Thus, academic partners may need to consider educational efforts on health, science, research, and digital literacy. Lastly, traditional researchers may be uncomfortable and unfamiliar with CBPR research. The strategies and tools used for CBPR research often differ from traditional clinical research and require a degree of flexibility with data capture. As community members are engaged in endeavors, special consideration for human subjects and data protections is required. Consequently, CBPR educational efforts, possibly co-designed with community members, and CBPR training and support for researchers is needed. Further, recognition that some researchers may not be well-suited to work directly with community and options for other ways these researchers could leverage existing data and samples would be essential.

## Conclusions

In summary, numerous studies benefit from an institutional biobank such as the GCC. Availability of appropriate controls with existing phenotype data as well as a wide variety of available samples purposefully collected can (1) substantially lower cost and time, and (2) inspire and enable unique exploratory efforts. While the population-based sampling employed in the GCC enabled broad utilization, partnering with communities to establish research registries could provide a cost effective mutually beneficial resource.

## Data Availability Statement

The datasets generated for this study are available on request to the corresponding author.

## Ethics Statement

The studies involving human participants were reviewed and approved by Cincinnati Children's Institutional Review Board. Written informed consent to participate in this study was provided by the participants' legal guardian/next of kin.

## Author Contributions

LMa conceived the study, performed the analyses, interpreted the data, and drafted the manuscript. LMu interpreted the results and provided critical revision. MBK conceived the study, interpreted the results, and provided critical revision.

## Conflict of Interest

The authors declare that the research was conducted in the absence of any commercial or financial relationships that could be construed as a potential conflict of interest.

## Supplementary Material

Supplementary Figure 1 Demographic comparison of the GCC to US census data.
