# Supplementary figures and images for: Building a Population Representative Pediatric Biobank: Lessons Learned From the Greater Cincinnati Childhood Cohort
Source: Front Public Health. 2021 Jan 14;8:535116. doi: 10.3389/fpubh.2020.535116 (PMC7841396; doi:10.3389/fpubh.2020.535116)

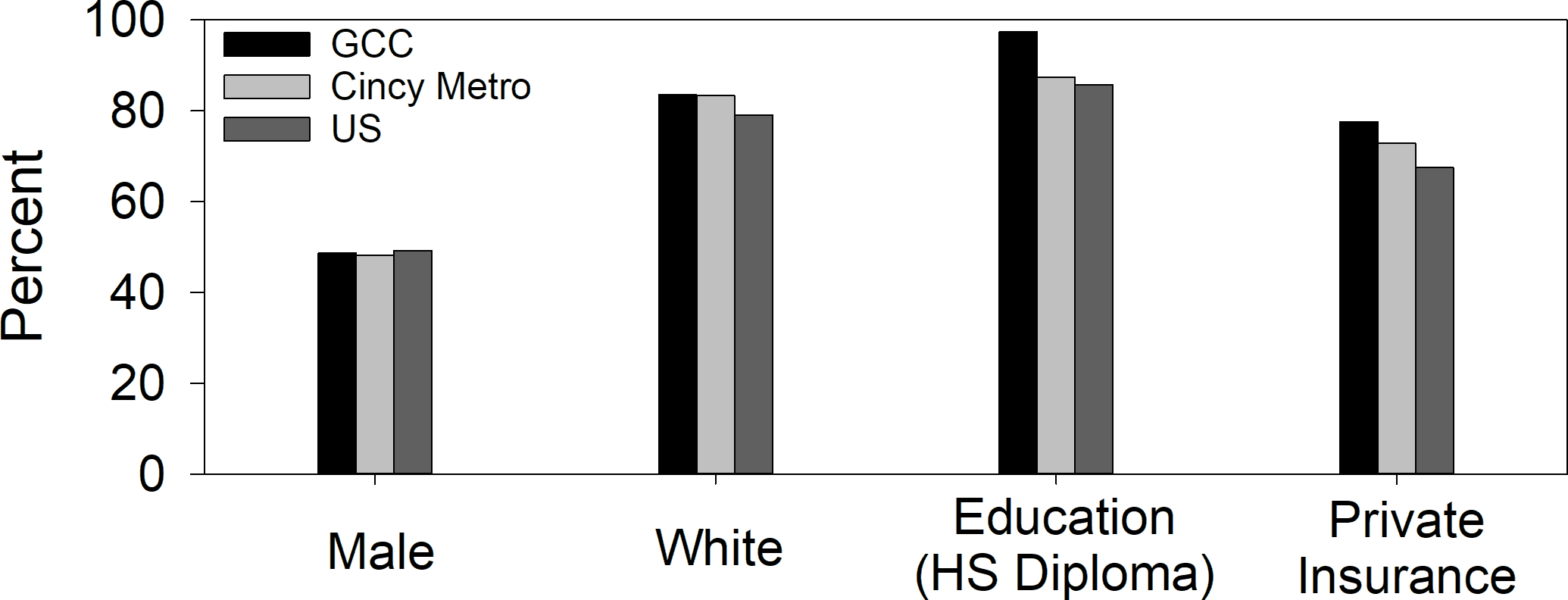

Supplement: Supplementary Figure 1 — Demographic comparison of the GCC to US census data. [file Image_1.TIF]
